# Type II Toxin–Antitoxin Systems in the Unicellular Cyanobacterium *Synechocystis* sp. PCC 6803

**DOI:** 10.3390/toxins8070228

**Published:** 2016-07-21

**Authors:** Stefan Kopfmann, Stefanie K. Roesch, Wolfgang R. Hess

**Affiliations:** Faculty of Biology, Institute of Biology 3, Genetics and Experimental Bioinformatics, University of Freiburg, Schänzlestr. 1, D-79104 Freiburg, Germany; stefan.kopfmann@gmx.de (S.K.); steffi-roesch@gmx.de (S.K.R.)

**Keywords:** bacterial toxins, cyanobacteria, hydrogenase, PIN-domain, cibonuclease, RNA degradation, RNA interferase, RNA turnover, toxin–antitoxin, VapC

## Abstract

Bacterial toxin–antitoxin (TA) systems are genetic elements, which are encoded by plasmid as well as chromosomal loci. They mediate plasmid and genomic island maintenance through post-segregational killing mechanisms but may also have milder effects, acting as mobile stress response systems that help certain cells of a population in persisting adverse growth conditions. Very few cyanobacterial TA system have been characterized thus far. In this work, we focus on the cyanobacterium *Synechocystis* 6803, a widely used model organism. We expand the number of putative Type II TA systems from 36 to 69 plus seven stand-alone components. Forty-seven TA pairs are located on the chromosome and 22 are plasmid-located. Different types of toxins are associated with various antitoxins in a mix and match principle. According to protein domains and experimental data, 81% of all toxins in *Synechocystis* 6803 likely exhibit RNase activity, suggesting extensive potential for toxicity-related RNA degradation and toxin-mediated transcriptome remodeling. Of particular interest is the Ssr8013–Slr8014 system encoded on plasmid pSYSG, which is part of a larger defense island or the pSYSX system Slr6056–Slr6057, which is linked to a bacterial ubiquitin-like system. Consequently, *Synechocystis* 6803 is one of the most prolific sources of new information about these genetic elements.

## 1. Introduction

Toxin–antitoxin (TA) systems are small genetic elements composed of a stable toxic protein and its unstable cognate antitoxin. They are encoded on chromosomes as well as on episomal genetic elements and are widely distributed throughout the prokaryotic domain of life. Many functions have been assigned to TA modules, ranging from plasmid maintenance to persister cell formation and stress response (for reviews, see [1,2,3,4,5,6,7,8]). TA systems exist in surprisingly high numbers in all prokaryotes and a growing number of studies suggest TA systems with milder effects to act as mobile stress response systems which help certain cells of a population in persisting adverse growth conditions. In general, the toxin is counteracted by its cognate antitoxin unless certain circumstances, such as plasmid loss or environmental stress, disrupt the TA balance and consequently frees the toxin to target various cellular functions. Whereas the toxin is always a protein, both proteins as well as RNA molecules have been found as antitoxins.

TA systems currently are classified into six types, based on the molecular nature of the antitoxins and the mechanisms by which they counteract the cognate toxins. Type I and type III are characterized by a small non-coding RNA (sRNA) as the antitoxin [9,10,11], whereas types II, IV, V and VI feature antitoxic proteins [1,12,13,14,15]. From these, Type II TA systems are certainly the best characterized systems with thousands of identified loci (reviewed in references [2,4,6]).

In this work, we focus on the unicellular cyanobacterium *Synechocystis* sp. PCC 6803 (from here: *Synechocystis* 6803), a model in biotechnology and fundamental research. DNA replication and metabolism, translation and cell membrane organization are among the verified cellular targets of bacterial TA systems. In cyanobacteria, oxygenic photosynthesis and thylakoid membranes appear as attractive additional targets, but very few cyanobacterial TA system have been characterized thus far. The numerically dominating TA systems belong to the Type II TA class. Makarova et al. (2009) [3] identified a total of 29 Type II TA pairs in *Synechocystis* 6803, whereas the TA Database [16,17] listed a total of 36 TA pairs, which are distributed over the chromosome and the four larger plasmids (Chr: 19; pSYSM: 5; pSYSX: 2; pSYSA: 6; pSYSG: 4). From these, only four chromosomally located TA pairs [18,19,20,21], one pair on plasmid pSYSX [22] and five located on plasmid pSYSA [23] were targeted experimentally thus far.

Addressing the number of possible Type II TA systems, here we expand the list of such putative systems in *Synechocystis* 6803 from 36 to 69 TA pairs and seven stand-alone TA components. From these 69 Type II TA loci, 47 are located on the chromosome and 22 are plasmid-located. With these numbers, *Synechocystis* 6803 is in the top-10 list of prokaryotic organisms with regard to the number of TA systems. We find that toxins belonging to one family are not always associated with antitoxins from a single family, as proposed in the classical classification system, but rather that different types of toxins are associated with various different antitoxins in a mix and match principle as proposed by Leplae et al. [4] and Makarova et al. [3]. According to the identified protein domains, 81% of all toxins in *Synechocystis* 6803 are predicted to exhibit RNase activity, suggesting extensive potential for toxicity-related RNA degradation but potentially also for toxin-mediated transcriptome remodeling. Indeed, the molecular characterization of 16 of these loci revealed various types of RNA endonuclease activities.

## 2. Results

### 2.1. Global Characterization of *Synechocystis* 6803 Type II TA Systems

Our results obtained in the course of this work indicated that TA systems in *Synechocystis* 6803 are much more abundant and diverse than previously thought. To identify undetected type II TA loci in *Synechocystis* 6803, we examined its genome and plasmids using the RASTA system [24], leading to the identification of seven new TA loci, which were not addressed in the TA database [17]. To extend this automated search, we scanned for putative TA systems based on the presence of known toxin and antitoxin associated protein domains [3,4,16,25,26]. We retrieved the gene IDs of *Synechocystis* 6803 protein sequences containing these domains via the Pfam database [27] and searched in a case-by-case analysis the genomic neighborhood of the predicted toxins or antitoxins for potential cognate TA partners. This led to the correction of six open reading frames by adjusting the 5′ ends according to multiple sequence alignments and information on the transcriptional start sites (TSSs; information extracted from references [28,29]) and the identification of seven novel TA-related open reading frames that had previously not been annotated in *Synechocystis* 6803 at all (Table 1).

**Figure 1:**
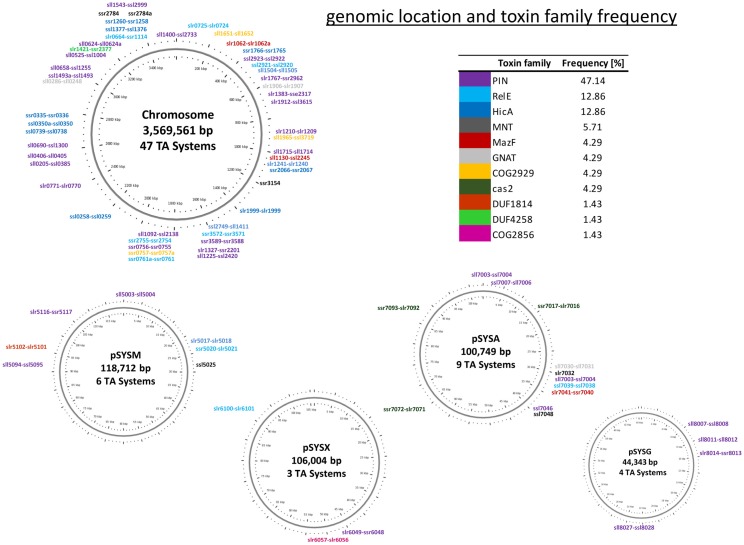
Frequency and distribution of Type II TA families in *Synechocystis* 6803. The classification of the 69 TA loci is based on the respective toxin component and given in %. The location of these loci over the chromosome and the four large plasmids is shown on the genomic maps. The color code of the TA loci matches the color code in the tabular overview. The length of plasmid pSYSA is according to reference [32]. Here, the three *cas1-cas2* gene pairs on plasmid pSYSA were not counted.

We therefore decided to classify the 69 TA loci in *Synechocystis* 6803 based on the putative toxin component. We observed that the *vapC*, *relE* and *hicA* families appeared most frequent, while *mazF* is under-represented in *Synechocystis* 6803 compared to other bacteria (Figure 1).

A special situation exists with the Cas1-Cas2 module, which encodes the only conserved proteins among all subtypes of the CRISPR–Cas system and is typically connected to spacer acquisition [30]. While Cas2 is a homolog of the VapD toxin and exhibits sequence-specific nuclease activity [26,31], Makarova et al. [26] proposed that *cas1*-*cas2* could act as a TA module and is therefore included here (Table 2).

**Figure 2:**
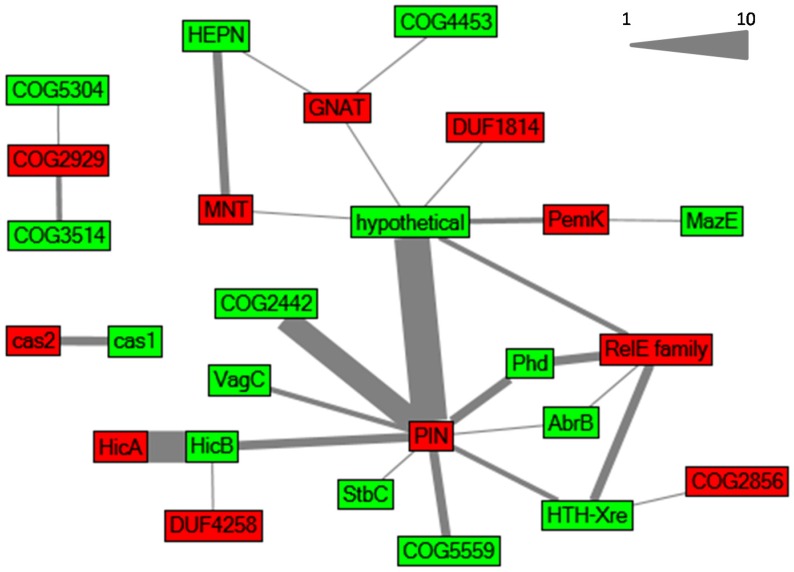
Relationships between different toxin and antitoxin families in *Synechocystis* 6803, illustrated in a network graph inspired by Makarova et al. [3]. Toxin and antitoxin families are depicted in red and green, respectively. The edges connect toxin and antitoxin families that were identified as cognate toxic and antitoxic components organized in a putative TA operon. The thickness of an edge is proportional to the abundance of the toxin–antitoxin combination. The scale represents the correlation between operon abundance and line thickness.

Toxins belonging to one family are not always associated with antitoxins from a single antitoxin family, as proposed in the classic classification system, but rather TA loci are formed in a mix and match principle [3,4]. We also observed this relationship (Figure 2).

### 2.2. Verification of Predicted TA Functionality

**Figure 3:**
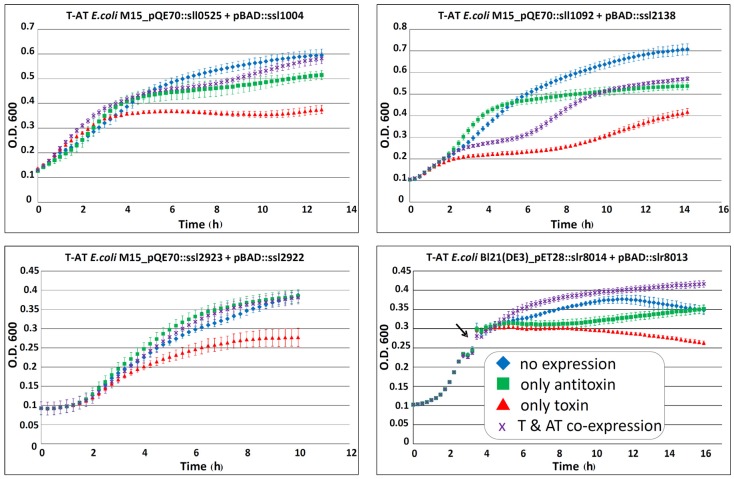
Effect of potential cyanobacterial toxin and antitoxin overexpression on growth of *E. coli* M15(pREP4) and Bl21(DE3) Rosetta™ harboring the plasmids pQE70::toxin or pET28a(+)::toxin and pBAD::antitoxin. Multiple TA systems were tested: genes *sll0525–ssl1004*; *sll1092–ssl2138*; and *ssl2923–ssl2922*; *slr8014–slr8013*. Expression of the potential toxins causes growth inhibition in the expression strains (*red triangle*), which can be prevented by co-expression of the respective antitoxin (*purple* *crossed*). Strains without expression are indicated by the *blue diamond lines*, and lone expression of antitoxin is indicated by the *green* *square lines*. The experiments were performed in duplicates or triplicates, and standard deviations are indicated. *T-AT*, toxin–antitoxin; *O. D.*, optical density; *T & AT co-expression*, toxin and antitoxin co-expression. For studying *slr8014–slr8013*, the culture was grown in LB medium to an optical density (O. D.) of ~0.3 and then split (indicated by the black arrow). Subsequently, the effect of lone toxin expression (*red triangle),* antitoxin expression (*green* *square lines),* co-expression (*purple* *crossed*) and the untreated control (*blue diamond*) was measured as described above.

To test the validity of predictions presented in Table 2, we selected four toxins for their ability to cause growth inhibition in *E. coli* that becomes attenuated by the associated antitoxin. We chose three chromosomally encoded TA systems and one located on plasmid pSYSG. To this aim, we expressed the toxin and antitoxin from different inducible promoters in *E. coli*. The different *E. coli* strains containing both pQE70::toxin (expression controlled by the addition of IPTG) and pBAD::antitoxin (expression controlled by the addition of arabinose) were grown to the required optical density in LB broth plus the respective antibiotics. The cultures were diluted 100-fold in fresh LB (plus antibiotics and the corresponding inducer) and the effects of separate toxin, antitoxin or toxin–antitoxin co-expression were analyzed by measuring the optical density over time in intervals of 15 min. Each of the four tested strains showed an inhibition in bacterial growth when the toxin was expressed alone, compared to strains with no expression or lone expression of the antitoxin (Figure 3). Co-expression of toxin and antitoxin attenuated the growth inhibitory effect for all four systems (Figure 3). Therefore, we conclude that the four tested systems exhibited typical properties of a TA system in vivo.

Hence, growth inhibition by the respective toxin and corresponding attenuation by the antitoxin has been demonstrated for altogether 11 *Synechocystis* 6803 TA systems: five located on plasmid pSYSA were previously analyzed [23], one located on plasmid pSYSG in this work (Figure 3) and five chromosomal TA systems analyzed before [18,20] or in this work (Figure 3).

### 2.3. *Synechocystis* 6803 Type II TA Systems of Special Interest

These analyses demonstrated interesting features of several of these previously known or newly defined Type II TA systems alike.

The *slr1767–ssr2962* TA system genes are located in an excludon-like arrangement in the genome, in which they are covered over their entire length by a long transcript in antisense orientation (TU520 [29]) that otherwise represents the mRNAs of genes *sll1639–sll1641* encoding the urease accessory protein UreD, nitrilase and glutamate decarboxylase. The excludon concept describes an unusual long asRNA, which acts as an asRNA regulator for one ORF while simultaneously comprising the ORFs and UTRs of the neighboring divergently oriented genes [34]. Furthermore, genes or operons organized in this arrangement often possess mutually exclusive or related functions [34]. All genes encoded in TU520 are involved in nitrogen metabolism, whereas the biological function of the TA module is unknown, but possesses a significant TSS only under nitrogen depletion [29]. According to the excludon concept [34], enhanced expression of the *slr1767–ssr2962* dicistron might lead to operon discoordination and lowered expression of the glutamate decarboxylase gene compared to the first two genes within TU520. The Slr1767–Ssr2962 TA system is functional, and has previously been characterized as a bona fide TA system [20]. However, while this is the first report of a TA system in such an arrangement, the comparison to the closely related strain *Synechocystis* 6714 revealed the homologs of the genes *slr1639–slr1641* to be conserved and within one operon, while the TA locus was missing. Thus, the *slr1767–ssr2962* dicistron has been inserted or deleted in one of the two strains. It has to be evaluated whether the *slr1767–ssr2962* TA module has a true biological function related to its excludon-like structure.

Another interesting and previously unrecognized variation of type II TA systems is the toxin–antitoxin fusion into a multi-domain protein. Such a situation was described with the EzeT system in *E. coli* in which the C-terminal domain of this type II TA system harbors a zeta-toxin like UDP-*N*-acetylglucosamine kinase domain that interferes with peptidoglycan synthesis, whereas the N-terminal domain harbors the antitoxin [35]. In *Synechocystis* 6803, we identified with Slr1999 an unusual putative TA component, consisting of a toxin (HicA) as well as an antitoxin (HicB) domain within one protein (Table 2). This fused protein probably resulted from the gene fusion of a conventional bicistronic operon, especially because there is no fused homolog detectable in any other organism. Another interesting aspect is that such a covalent linkage between toxin and antitoxin into a single polypeptide should lead to an auto-regulated toxin.

### 2.4. *Synechocystis* 6803 Type II TA Systems in the Maintenance of Plasmids and Genomic Islands

One of the proposed functions of TA systems is the maintenance of plasmids and genomic islands through the post-segregational killing mechanism. Furthermore, TA modules can be classified as anti-phage defense systems due to their ability to mediate dormancy and even cell death during phage attack [36,37,38]. In general, defense and TA systems can be found enriched in defense islands and on account of their addictive properties also in mobilome islands [39]. We inspected the localization and the genetic neighborhood of the putative TA loci to determine their potential association with plasmid, or genomic, defense and mobilome islands stabilization. All four large plasmids harbor at least three putative TA systems (Figure 1). However, the plasmid pSYSA encodes even ten different TA modules (one potential Type I, six Type II and three Cas1/Cas2) plus three stand-alone components.

#### 2.4.1. The Slr6057–Slr6056 TA System May Stabilize a Genetic Cassette for a Bacterial Multiubiquitin System on Plasmid pSYSX

**Figure 4:**
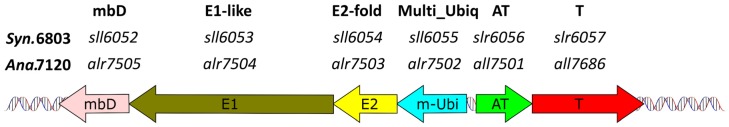
Genetic organization of a likely bacterial multiubiquitin and functionally associated genes in connection to a TA system on plasmid pSYSX of *Synechocystis* 6803 (Sy.6803), compared to a similar system in *Anabaena* (*Nostoc*) sp. PCC 7120 (Ana.7120). mbD, metal binding domain; E1, functionally related to ubiquitin activating enzyme E1; E2, functionally related to ubiquitin transferring enzyme E2; m-Ubi , multiubiquitin; AT, antitoxin; T, toxin.

On plasmid pSYSX, *Synechocystis* 6803 possesses a cassette for a possible bacterial multiubiquitin system. The ubiquitin protein modification system is common to all eukaryotes, but is only rarely found in bacteria or archaea [40,41]. However, bacterial homologs to ubiquitin and the components of the ubiquitin modification system (E1, E2) have been found in divergent bacteria and archaea [42]. In *Synechocystis* 6803, these components are encoded on the plasmid pSYSX in a typical arrangement (multiubiquitin; E2; E1; metal binding protein) described by Iyer et al. [42], adjacent to the *slr6057–slr6056* locus (Figure 4).

There are no homologs of this system nor divergent ubiquitin-like gene families in the closely related *Synechocystis* 6714, making this cassette likely to have originated from lateral gene transfer. Consistent with this idea, within cyanobacteria a homolog was detected only in distantly related species such as *Pseudanabaena* sp. PCC 7367, *Oscillatoria nigro-viridis* or *Anabaena (Nostoc)* sp. PCC 7120 (genes *alr7502..alr7505*) on plasmid beta [42], which were also located adjacent to a homologous TA locus (Figure 4). Therefore, we extended our screen to the genetic neighborhood of all genes in the Pfam database [43], coding for bacterial multiubiquitin domain (PF14452) proteins and their vicinity to TA modules. We observed that 70% of these proteins were adjacent to potential TA loci, in which all toxic components were characterized by the same COG2856/DUF955 domain we found in the Slr6057 toxin. Thus, a stabilization effect caused by the Slr6057–Slr6056 TA locus on this bacterial multiubiquitin cassette appears likely.

#### 2.4.2. Is the hoxEFUYH Hydrogenase Operon in *Synechocystis* 6803 Stabilized by a TA System?

Our previous transcriptomic analyses [28,29] revealed that not all TA loci become transcribed from their own distinct TSS but some are part of a longer TU. Likewise, the Sll1225–Ssl2420 TA module and the TA associated DUF820-domain protein Sll1222 are encoded within the *hox* hydrogenase operon (*hoxEFUYH*). This operon is of special interest from a biotechnological perspective, since the bidirectional hydrogenase encoded by the *hox* operon could be used for biohydrogen production [44]. A very similar gene arrangement exists in *Synechocystis* 6714, where in extension of the conserved *hoxEF_sll1222_hoxUY_sll1225_ssl2420_hoxH* gene order, the TA system genes are interspersed with another hydrogenase-related gene, *hypD* (D082_26330), encoding the NiFe hydrogenase metallocenter assembly protein HypD, whereas the homologue in *Synechocystis* 6803, *slr1498*, is located 1.62 Mb away [45]. In contrast; other cyanobacteria seldom encode the *hox* operon in a coherent gene cluster according to the information obtained from the cyanobacterial genome database CyanoBase [46]. However, we identified in seven other strains TA modules or DUF820-domain proteins in the vicinity of some corresponding *hox* genes. In most cases, TA modules or DUF820-proteins clustered with the *hoxEF* operon, encoding part of the HoxEFU diaphorase subunit in cyanobacteria, which catalyzes the oxidation/reduction of NAD(P)H/NAD(P) [47]. HoxE can functionally be substituted by an unrelated subunit (HoxI) in other bacteria [47], but was shown to be an essential component for the connection between the NAD^+^/NADH and H^+^/H_2_ oxidoreduction reactions in *Synechocystis* [48]. In connection with the frequent clustering of TA systems and the *hox* operon, especially the *hoxEF* subunit, the question arises whether TA modules stabilize the whole or part of the hydrogenase *hox* operon. We consider the stabilization of the whole *hox* operon in *Synechocystis* as likely.

#### 2.4.3. Two Type II TA Systems Are Part of the Large 40 kb Genomic Island in *Synechocystis* 6803

The *slr1062–slr1062a (norf2)* encoding a PemK-type TA system is central to TU355. This TU is with 22 genes transcribed into a single consecutive mRNA the longest multicistronic transcript in *Synechocystis* 6803 [29]. TU355 and the neighboring region comprise mainly genes usually not found in other cyanobacteria. Among the encoded functions are enzymes for the modification of cell surface structures and rfb-glycosyltransferases. The transcription into long consecutive mRNAs and the fact that these genes have no close homologs, even in the otherwise closely related *Synechocystis* sp. PCC 6714 [45] suggest that it is a genomic island. Moreover, there is with the gene pair *ssr1765–ssr1766* a second Type II TA system in this region. Therefore, the location of *slr1062–slr1062a* and of *ssr1765*–*ssr1766* is consistent with the idea that these stabilize this genomic island.

### 2.5. RNase Activity Is the Most Frequent Characteristics Associated with Type II TA Systems in *Synechocystis* 6803

**Figure 5:**
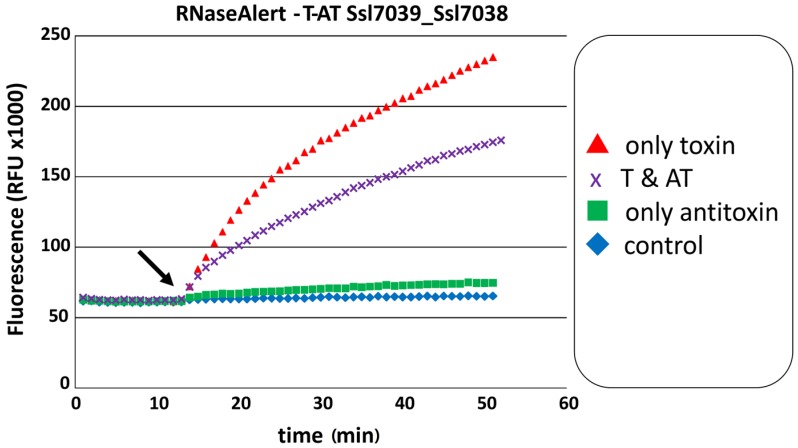
The RelE domain protein Ssl7039 exhibits ribonuclease activity, which is significantly hampered by antitoxin Ssl7038. The RNase Alert™ nuclease detection system was used. Fluorescence is indicated as function of time. The addition of the toxin Ssl7039 (arrow) shows a significant increase in fluorescence (*red triangle*) as compared with the buffer control (*blue diamond*). The simultaneous addition of toxin and antitoxin (arrow) results in a significantly lower increase in fluorescence (*purple crossed*). RFU, relative fluorescent units. The experiment was repeated independently (see also Figure 6).

According to the protein domains assigned to the here identified 69 Type II TA loci in *Synechocystis* 6803, 81% of all toxins are predicted to exhibit RNase activity, suggesting extensive potential for toxicity-related RNA degradation but potentially also for toxin-mediated transcriptome remodeling. To test this prediction experimentally, several of the predicted toxins were purified and their potential ribonuclease activity assayed. Initial results indicated a ribonuclease activity for the putative toxins Ssl2923 encoded on the chromosome, Slr5116 encoded on plasmid pSYSM, Ssl7007 and Ssl7039 encoded on plasmid pSYSA, and Sll8011 encoded on plasmid pSYSG. These proteins possess a PIN domain (Ssl2923, Slr5116, Ssl7007, Sll8011) or a RelE domain (Ssl7039). Furthermore, the tested toxins encoded on plasmid pSYSA Sll7003 and Ssl7039, exhibited RNase activity ([23] and Figure 5).

#### 2.5.1. The RelE Protein Ssl7039 Is a Ribosome-Independent Ribonuclease in Vitro and Leads to the Degradation of *E. coli* lpp mRNA in Vivo

Co-expression of the respective toxin and antitoxin in vivo showed for multiple *Synechocystis* 6803 TA systems that the growth inhibition effect of the toxin was attenuated by the respective antitoxin ([18,20,23] and Figure 3). Therefore, we tested if the proposed toxic RNase activity of a purified toxin is abolished by the purified antitoxin. For this experiment, we chose the previously not studied Ssl7039–Ssl7038 TA system. RNase activity of Ssl7039 can be seen by a rapid increase of fluorescence over time after addition of the toxin, compared to an even level of fluorescence in the buffer and Ssl7038 antitoxin control. Simultaneous addition of both toxin and antitoxin resulted in a significant lower increase of fluorescence (Figure 5).

**Figure 6:**
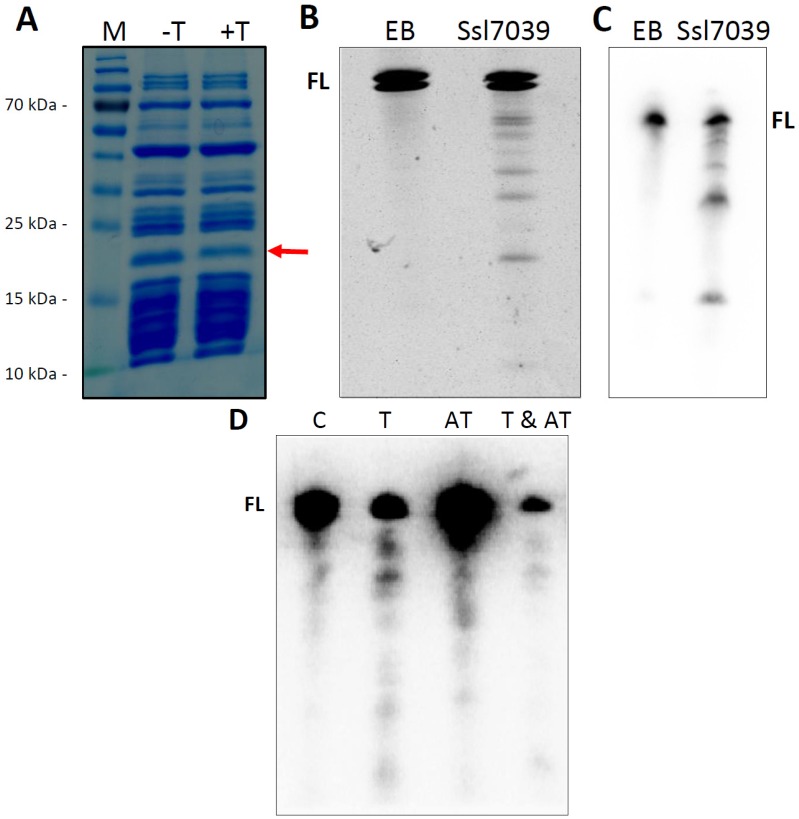
Purified toxin Ssl7039 exhibits ribosome-independent RNase activity. (**A**) Addition of Ssl7039 to the coupled transcription-translation approach, expressing the DHFR control template, decreases DHFR protein abundance. The DHFR band is labeled by an arrow. The synthesis reaction were separated on 10% SDS–PAA gel electrophoresis and visualized by Coomassie staining. M, marker (PageRuler, Thermo Fisher Scientific); -T, no addition of purified Ssl7039; +T, addition of purified Ssl7039. (**B**) In vitro synthesized *dhfr* mRNA was incubated for 30 min with purified toxin Ssl7039, resulting in a clear cleavage pattern. Incubation with elution buffer (*EB*) served as control. The reaction were separated on 6%–10% 7 M urea polyacrylamide gels (6 mA, 1.5–2.5 h) and visualized by ethidium bromide staining. FL, full length *dhfr* mRNA (~500 nt). (**C**) Total RNA from the coupled transcription/translation approach was extracted 2 h after toxin addition, followed by northern analysis targeting the *dhfr* mRNA. EB, elution buffer control; Ssl7039, addition of purified Ssl7039. (**D**) Expression of Ssl7039 decreases *lpp* mRNA stability in vivo. The expression strain *E. coli* Bl21 (DE3) Rosetta, harboring the plasmid pET28a(+)::ssl7039 and pBAD::ssl7038, was grown in LB medium to an optical density (O. D.) of 0.3–0.4 and then split before addition of the appropriate inducer. Total RNA was extracted 30 min after inductor addition, followed by northern analysis aimed at the *lpp* mRNA. FL, full length *lpp* transcript; C, no inducer; T, toxin expression; AT, antitoxin expression; T & AT, both toxin and antitoxin expression.

The RelE-family can be divided into five subfamilies, which show ribosome-dependent as well as ribosome-independent ribonuclease activity. The gp49-domain is assigned to the RelE-superfamily and has been suggested to represent a subclass of HigB [49]. The subfamily HigAB commonly features an inverted gene order and solely ribosome-dependent mRNA cleavage. In addition to the presence of a gp-49 domain in Ssl7039, the *ssl7038–ssl7039* locus also exhibits an inverted gene order. To determine if the toxin Ssl7039 is exclusively ribosome-dependent we tested the ribonuclease activity of the purified toxin using a cell-free transcription/translation system and in vitro transcribed RNA, respectively. Addition of the purified toxin to the coupled transcription/translation approach, expressing the DHFR control template, caused a decrease in the amount of DHFR protein (Figure 6A). We assayed the template mRNA stability, in order to evaluate if protein translation arrest was mediated by the endoribonuclease activity of Ssl7039. Total RNA was extracted and separated by PAGE. Northern analysis confirmed degradation of *dhfr* mRNA, resulting in several specific cleavage products, exclusively after toxin addition (Figure 6B). To investigate if this endoribonucleolytic activity was ribosome dependent, we utilized in vitro synthesized *dhfr* mRNA as substrate. Substrate RNA was incubated with purified toxin and analyzed by PAGE separation and ethidium bromide staining. Free *dhfr* mRNA was also cleaved into several fragments by the toxin, resulting in a distinct cleavage pattern similar to the coupled cell-free transcription/translation assay (Figure 6C).

To extend these results further, we checked the stability of the *lpp* mRNA in vivo in the *E. coli* M15(pREP4)_pQE70::ssl7039 + pBAD::ssl7038 expression strains. The *lpp* transcript was chosen for examination because it is highly expressed and was used in several toxin cleavage assays before [50,51,52].

Total RNA was extracted and separated by PAGE. Northern analysis revealed a decreased transcript level of the *lpp* mRNA exclusively after Ssl7039 induction compared to no or Ssl7038 induction (Figure 6D). In addition, distinct degradation products were detected only after toxin expression. In contrast, co-expression of toxin and antitoxin resulted in a decreased transcript level with no distinct degradation products (Figure 6D). Therefore, the *lpp* expression level was lowered for other (non-toxin mediated) reasons, like, e.g., the metabolic burden from co-expressing both proteins. We conclude that Ssl7039 is a RelE-type ribosome-independent ribonuclease.

#### 2.5.2. Heterologous Expression of Slr8014 in *E. coli* Causes Degradation of lpp mRNA in Vivo That Is Abolished by the Associated Antitoxin

**Figure 7:**
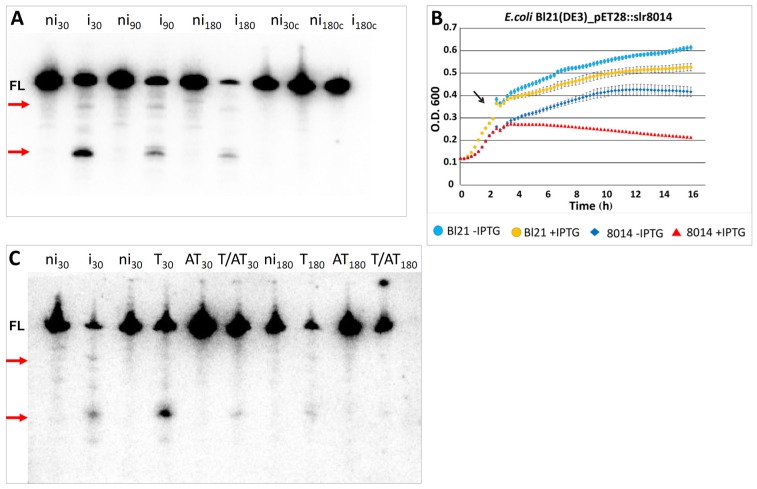
Expression of Slr8014 causes growth inhibition of *E. coli* and decreases *lpp* mRNA stability in vivo that is attenuated by the associated antitoxin. (**A**) Overexpression of the *Synechocystis* 6803 toxin Slr8014 in expression strain *E. coli* Bl21 (DE3) Rosetta harboring the plasmid pET28a(+)::slr8014. Total RNA was extracted at the indicated time points after IPTG addition, followed by northern analysis aimed at the *lpp* mRNA. Cleavage products are labeled by red arrows. FL, full length *lpp* transcript; ni, no IPTG induction; i, IPTG induction; ni_c_ + i_c_, negative controls (Bl21 (DE3) Rosetta strain harboring no plasmid). (**B**) Expression of the *Synechocystis* 6803 toxin Slr8014 causes strong growth inhibition in the expression strain *E. coli* Bl21 (DE3) Rosetta harboring plasmid pET28a(+)::slr8014 when the inducer IPTG was added (*red triangles*), but only a mild growth retardation when no IPTG is added (*blue diamonds*). Cultures continued to grow with and without IPTG addition in the no plasmid strain (*yellow and blue dots*). The experiments were performed in triplicates, and standard deviations are indicated. *T-AT*, toxin–antitoxin; *O. D.*, optical density; *T & AT co-expression*, toxin and antitoxin co-expression. The culture was grown in LB medium to an optical density (O. D.) of 0.3-0.4 and then split (indicated by the black arrow). IPTG (final concentration 1 mM) was added to one-half (*red triangle*), and the other half was left untreated (*blue diamond*). Control treatment was analogously performed with the *E. coli* Bl21 (DE3) Rosetta expression strain harboring no plasmid. The experiments were performed in triplicates, and standard deviations are indicated. (**C**) Inhibition of the toxic RNase activity by co-expression in *E. coli*. Total RNA was extracted at the indicated time points after IPTG addition, followed by northern analysis aiming at *lpp* mRNA. Cleavage products are labeled by arrows. FL, full length *lpp* transcript; ni, no IPTG induction; i, IPTG induction; T, only toxin; AT, only antitoxin; T&AT, toxin and antitoxin co-expression.

We evaluated the potential RNase activity of the heterologously expressed toxin Slr8014 in vivo*.* For this aim, we performed northern analysis on *lpp* mRNA stability after toxin induction in the *E. coli* Bl21 (DE3) Rosetta pET28a(+)::slr8014 expression strain (Figure 7). Northern analysis showed time dependent *lpp* mRNA decay, which resulted in a prominent small and a faint large cleavage product compared to the non-induced strain (Figure 7A). Parallel growth inhibition assays showed that, compared to the strain with no expression, lone induction of Slr8014 caused significant growth inhibition (Figure 7B).

To extend these results further, we examined whether the cognate antitoxin Ssr8013 was able to neutralize the toxic Slr8014/VapC activity. The co-expression of toxin and antitoxin attenuated both the growth inhibitory effect (see Figure 3) and toxin RNase activity. Lone expression of antitoxin did not influence *lpp* mRNA integrity at any measured time. Co-expression of toxin and antitoxin led to increased *lpp* stability (Figure 7C). Compared to the solely expressed toxin, the prominent cleavage product was hardly seen 30 min past induction. Additionally, we observed a significant decrease of full length *lpp* mRNA after 180 min IPTG induction (Figure 7C).

## 3. Discussion

Here we present the comprehensive analysis of Type II TA systems in the cyanobacterial model organism *Synechocystis* 6803. Altogether, we define 76 likely Type II TA systems, roughly doubling the previously known number. From these, 69 occur in the classical form of a TA gene pair and seven as stand-alone components. We suggest three loci to be translated from leaderless mRNAs and seven to be associated with an asRNA. Altogether, 16 of the 69 two-component loci have been analyzed thus far by biochemical or molecular genetic approaches. The experimentally obtained results are consistent with the prediction that RNase activity is the major reason for the toxicity of these TA systems. In fact, based on the domain annotation it appears likely that 57 of the defined elements possess RNase activity.

### 3.1. Transcriptional Features of *Synechocystis* 6803 Type II TA Systems

Comparative analysis of the *Synechocystis* 6803 primary transcriptome established genome-wide TSS maps and consequently transcriptional units under different, environmentally relevant stimuli [28,29]. According to the criteria defined in these publications almost all of the putative TA pairs are transcribed, and distinct TSS could be identified for the majority of TA loci (Table 2).

We noticed that the TSS of the *sll1651*, *sll1965* and *ssl7039* toxin genes coincide with their respective start codons, pointing to their possible leader-less initiation of translation. Leader-less initiation of translation has been associated with the stress-dependent expression of certain genes [53,54]. Intriguingly, all TA loci affiliated to leader-less initiation of translation in *Synechocystis,* exhibit a reverse gene order.

This TSS data examination revealed furthermore the presence of internal TSSs within the 5′ segment of the coding region of the leading TA operon element in some cases. Based on these TSSs we suggest in this work possible alternative start codons (Table 1). In addition we noticed with 207 nt a quite long 5′ UTR for the *sll7006–ssl7007* module. Moreover, the annotated start of *ssl7007* is an alternative start codon (GTG), and there is not a single in frame stop codon within the 5′ UTR [23]. In general, blastp analysis of the possible translation products of the shortened or extended open reading frames revealed a very high degree of conserved residues with predicted protein sequences from several other cyanobacteria. Blast analysis of Ssr0756 also identified only an incomplete toxin domain, which could be completed by extending the ORF starting with an alternative start codon (Table 1). The prolonged open reading frame was further supported by the comparison of *Synechocystis* 6803 and 6714 homologs. We conclude that the genes listed in Table 1 should be re-annotated accordingly.

Type I TA systems are defined by their small non-coding RNA antitoxins, which are often located antisense to their cognate toxin. Genome-wide TSS mapping suggested massive antisense transcription in *Synechocystis* 6803 [28,29,55], consistent with the likely existence of multiple putative type I TA systems. Notwithstanding this possibility, we also detected seven Type II TA modules associated with antisense-located transcriptional units (Table 2). Usually, Type II TA systems are organized in a bicistronic operon and the transcriptional control is typically secured by autoregulation. Therefore, this organization could depict an additional regulatory element in Type II TA system control. Besides a possible antisense regulatory element we found other conspicuous features not commonly described for TA systems. We detected the putative TA systems *sll0624a–sll0624*, which exhibited mapped TSS for both the antitoxin and toxin component. On the other hand, the *ssl0259*–s*sl2058* loci showed only a TSS for the subsequent antitoxin gene, whereas no TSS could be found for the leading toxin gene (Table 2). Three TA modules (*ssl1300–sll0690*; *ssr0761–ssr0761a*; and *ssr2962–slr1767*) held two alternative TSSs, indicating potential alternative transcripts (Table 2). For instance, the TA pair *sll0690*–s*sl1300* displayed two TSSs which are differentially regulated under diverse environmental stimuli, showing the highest expression levels under heat stress and in the absence of light, respectively. Another eleven (*ssl0350–ssl0350a*; *slr0770–slr0771*; *slr1062a–slr1062*; *slr1209–sl1210*; *ssl2420–sll1225*; *ssr1258–ssr1260*; *ssl2733–sll1400*; *ssr2067–ssr2066*; *ssl2920–ssl2921*; *ssl2922–ssl2923*; and *ssr3588–ssr3589*) TA modules do not possess a distinct TSS, but are part of a larger TU (Table 2).

### 3.2. Similarities of *Synechocystis* 6803 Type II TA Systems to Those of Other Cyanobacteria

Based on their association with known mobile elements [56] or their frequently scattered distribution, TA systems are often considered as highly mobile genetic elements and, in many cases exist only in some isolates of a given species or genus [57,58,59]. Comparing the suite of *Synechocystis* 6803 Type II TA systems to those of *Synechocystis* 6714 (16S rDNA identity 99.4% [45,60]), we identified homologs for only 28% of toxin and 24% of antitoxin DNA sequences using blastn and a cut-off of 65% sequence identity. However, using blastp at a 30% cut-off of sequence identity, we detected 47% toxin and 37% antitoxin homologs. Furthermore, toxins and antitoxin are often small proteins defined by a single domain and we noticed that in some cases one and the same sequence had the same corresponding blastp hit. It cannot be excluded that these TA loci depict paralog systems, but we narrowed each case down to a single ortholog using reciprocal blastp analysis and synteny analysis of the genetic neighborhoods in strains 6803 and 6714, resulting finally in a number of 40% orthologous toxins and antitoxins, respectively (Supplementary Materials Tables S1 and S2). Kopf et al. [45] observed that approximately 77% of the *Synechocystis* 6803 protein-coding genes share homologs in *Synechocystis* 6714. Consequently, both strains share significantly less TA loci compared to the total number of shared protein coding genes, indicating a high mobility of the TA modules. This result is supported further by the observation that at least twelve TA loci insertion/deletion events happened for complete TA systems or components in one of the two strains. Due to their mobility, Type II TA systems are thought to move from one genome to another by horizontal gene transfer, resulting in a great variation of TA loci among bacterial genera and isolates [4]. As seen in the comparison of the closely related *Synechocystis* strains 6803 and 6714, several TA loci were not found in strain 6714, indicating insertion/deletion events or an alien origin. For instance, the blastp analysis of the *slr1062a–slr1062* TA system located within the ~40 kb genomic island resulted in top-matching hits in non-cyanobacterial species and was most closely related to proteins in *Thiocapsa marina* (67% identity) and *Halomonas* sp. TG39a (62% identity), respectively. This diversity was observed for the complete island, pointing to an alien origin of the genomic region [45] stabilized by TA systems.

Furthermore, blastp analyses of *Synechocystis* 6803 TA loci against other cyanobacterial species, sharing the same clade based on 16S rRNA phylogeny [61], and the distantly related *Synechocystis* sp. PCC 7509 indicated a high variation of orthologous TA systems in these species. Based on the toxin sequence, seven orthologs were found in all closely related strains (*Sll0205*; *Sll0286*; *Ssr0335*; *Ssl0350a*; *Slr1327*; *Sll0690*; and *Slr1906*), whereas the TA systems *sll0406–sll0405* and *sll5003–sll5004* were not found in any other strain. However, in contrast to the situation for Slr1062–Slr1062a, the top-matching proteins for Ssl0406 and Sll5003 belonged to the cyanobacteria *Synechococcus* sp. PCC 7335 and *Gloeocapsa* sp. PCC 73106, respectively. TA systems occurring in all examined strains could be of special interest since the orthologs were restricted to cyanobacteria and absent from bacteria such as *E. coli* K-12 or *Mycobacterium tuberculosis* H37v.

### 3.3. *Synechocystis* 6803 Type II TA Systems with a Putative Function in the Maintenance of Plasmids and Genomic Islands

TA systems are frequently enriched on plasmids, in genomic islands and other mobile genetic elements [39] and this relation can also be seen in *Synechocystis* 6803. All four large plasmids harbor at least three putative TA systems and in the case of pSYSA even nine TA modules plus three stand-alone components (Figure 1). This extremely high number might in case of pSYSA be linked to its remaining functions: 75% of the pSYSA coding capacity encode three different CRISPR systems [33]. Besides the connection between the TA loci and the defense plasmid pSYSA, we observed the clustering of TA systems among each other, defense genes and mobile elements also on several other occasions. One potential defense island is located on plasmid pSYSG. This island encompasses 26 genes and is composed of three putative TA systems (*ssl8008–sll8007*; *sll8012–sll8011*; and *ssr8013–slr8014*), four genes associated with the type I restriction-modification system (*ssl8049*; *sll8006*; *sll8009*; and *ssl8010*); six mobile elements (*ssl8041*; *slr8042–slr8046*; and *sll8017)* and five genes related to TA modules (*sll8001*; *sll8002*; *ssl8003*; *sll8004*; *ssl8005*), which have not met our strict identification criteria. A possible mobilome island can be found on the chromosome, where three consecutive TA loci (*ssl2922–ssl2923*; *ssl2920–ssl2921*; and *sll1504–sll1505*) are interspersed between a partial transposase (*ssr2699*) and a mobile retron-type reverse transcriptase gene (*sll1503*).

Comparative genome analysis of the closely related *Synechocystis* strains 6803 and 714 revealed the occurrence of a large ~40 kb genomic island [45]. We examined whether the genomic island contained TA modules and noticed that two putative TA loci (*slr1062a–slr1062*; and *ssr1765–ssr1766*) were located within this region in *Synechocystis* 6803. It has to be mentioned that the here identified gene *slr1062a* has previously been annotated as a novel open reading frame (*norf2*) of unknown function on basis of transcriptomic evidence [28].

It is furthermore of high potential interest that functional gene cassettes, such as the bacterial multiubiquitin system on plasmid pSYSX or the chromosomally located hydrogenase operon appear to be stabilized by the presence of respective TA loci.

## 4. Conclusions

The comprehensive analysis of Type II TA systems in the cyanobacterial model organism *Synechocystis* 6803 revealed the presence of 76 likely Type II TA systems (including three CRISPR-Cas loci and seven stand-alone components). For comparison, analyzing the numbers and types of TA systems in the genomes of 10 different *Xanthomonas* strains, between five and 15 complete Type II TA loci were identified per strain [62], whereas the average total genome size in the genus *Xanthomonas* (~5 Mb [63]) is about one-third larger than *Synechocystis* 6803 (3.93 Mb, cf. Figure 1). The highest number of TA systems in TADB is predicted for the cyanobacterium, *Microcystis aeruginosa* NIES-843 with 113 loci [17]. *Microcystis* and *Synechocystis* are closely related genera. These numbers suggest that *Synechocystis* 6803 and similar cyanobacteria constitute one of the most prolific sources of new information about these genetic elements. From all Type II TA systems in *Synechocystis* 6803, 47 are encoded on the chromosome and altogether 57 were demonstrated or predicted to encode RNase activity. These two facts together suggest that in addition to the known functions, several of these systems could play a hitherto underestimated role in remodeling the transcriptome under certain conditions.

## 5. Materials and Methods

### 5.1. Comprehensive TA System Detection

Several sources were combined to identify novel Type II TA modules in *Synechocystis* 6803 in silico. First we employed the web-based identification tool RASTA-bacteria, an automated method allowing identification of TA loci in sequenced prokaryotic genomes, to predicted TA loci for the chromosome and all plasmids [24]. These loci were compared with *Synechocystis* 6803 TA modules listed in the TADB [16]. Additional predicted TA loci, with a probability score up to 50%, were evaluated case by case using blastp analysis and genetic neighborhood information. Furthermore, protein domains associated with TA systems were gathered by literature search [3,4,16,25,26,64,65,66,67]. Because of the differences between these programs, we also performed blastp searches (*E* < 10^−4^) in which all TA proteins were used as queries against the proteins from the studied *Synechocystis* genomes. Corresponding *Synechocystis* 6803 protein IDs, and eventually gene IDs, were retrieved from the Pfam database [43]. Again, potential TA components were evaluated by case to case analysis based on blastp and genomic neighborhood information. TA components were classified as part of a potential TA system, when a neighboring gene (up- or downstream) was located in close proximity and also featured a potential TA domain or was affiliated with TA systems. TA affiliation was evaluated based on the results of the blastp analysis and assigned when multiple homologous proteins were also adjacent to a TA component. Stand-alone TA components were identified by comparison with the genome sequence of the closely related strain *Synechocystis* 6714 [45,60] based on homology and genomic context. Information on TSSs was retrieved from their previous genome-wide mapping in *Synechocystis* 6803 [28,29] and *Synechocystis* 6714 [68].

### 5.2. Experimental Methods

#### 5.2.1. Growth Inhibition Assay

The growth conditions for studying growth inhibition by toxins are given in [23].

#### 5.2.2. Overexpression and Purification of Toxins and Antitoxins

The strains used for heterologous protein expression and primers for construction of expression plasmids (pQE70::Toxin, pET28a(+):Toxin, and pBAD::Antitoxin) are given in Tables S3 and S4. Overexpression of toxin (His)_6_ and antitoxin (His)_6_ was induced by adding IPTG (0.1 or 1 mM) for 3 h at 37 °C with shaking. Lysis and wash buffer composition was constant at 50 mM NaH_2_PO4, 300 mM NaCl, whereas best imidazole concentrations was experimentally tested for concentrations between 10 and 30 mM (lysis buffer) and 20–60 mM (wash buffer).

#### 5.2.3. In Vitro Synthesis of RNA

In vitro transcription of the *DHFR* mRNA was carried out using the control plasmid of the PURExpress^®^ In Vitro Protein Synthesis Kit (NEB) or from the PCR product using *dhfr* specific primers (Tables S3 and S4.).

#### 5.2.4. RNA Extraction

*E. coli* total RNA extraction was performed following Hein et al. [69] with certain modifications. Cultures were harvested through centrifugation (8000 rpm, 20 °C, 15 min), resuspended in 1 mL PGTX [70] and flash-frozen in liquid nitrogen. Samples were incubated for 15 min at 65 °C and cooled down on ice before 1 vol chloroform/isoamylalcohol (24:1) was added. Samples were incubated at room temperature for 10 min under repetitive agitation. Phases were separated by centrifugation (swing out rotor at 6000 rpm, 15 °C, 15 min) and the upper aqueous phase was transferred to a new vial. Again, 1 vol chloroform/isoamylalcohol (24:1) was added and phases separated as described above. The upper aqueous phase was transferred to a new tube and gently mixed with 1 vol isopropanol. RNA was precipitated overnight at −20 °C and pelleted through centrifugation (13,000 rpm, 4 °C, 30 min). The pellet was washed with 1 mL of 70% ethanol (13,000 rpm, 20 °C, 5 min), allowed to air dry and resuspended in an appropriate volume of water, depending on favored RNA concentration.

#### 5.2.5. RNase Activity

Detection of RNase activity was measured by fluorescence RNaseAlert™ essentially as described in Kopfmann et al. [23]. RelE toxin Sll7039 reaction buffer was at a final concentration composed of 25 mM Tris-HCl (pH 7.5).

Specific in vitro cleavage assays were performed as described previously [23]. Purified toxin and antitoxin, 100–250 ng of each, were incubated with 50–400 ng of target in vitro transcripts at 30 or 37 °C for 30–120 min in 25 mM Tris-HCl (pH 7.5), 60 mM KCl, 100 mM NH_4_Cl, 5 mM MgCl_2_, and 0.1 mM DTT and 25 mM Tris-HCl (pH 7.5) respectively.

In vivo *cleavage Assay*—*E. coli* BL21 (DE3): Rosetta pET28(a)::toxin strains and *E. coli* BL21 (DE3) pET28(a)::toxin + pBAD::antitoxin strains were inoculated and grown at 37 °C with shaking in LB medium containing appropriate antibiotics until an *A*_600_ 0.18–0.3 was reached. Strains were grown at 37 °C with shaking in LB medium for another 30–180 min upon IPTG and L-arabinose addition. Cultures expressing only toxin, only antitoxin, both proteins, or none of these protein were harvested by centrifugation (8000 rpm, 15 min), resuspended in 1 mL PGTX [70], immediately frozen in liquid nitrogen and stored at −80 °C. Total RNA was extracted as described above, followed by DNase treatment (2 units of Turbo DNase, Invitrogen) and phenol/chloroform purification. Five micrograms of treated total RNA per sample were separated on 10% polyacrylamide-urea gels, electroblotted onto Hybond-N+ membranes from Amersham and cross-linked via UV-light exposure. Northern analysis was performed following Stazic et al. [71]. Membranes were hybridized overnight at 57 °C in hybridization buffer (50% deionized formamide, 7% SDS, 250 mM NaCl, 120 mM NaPi buffer, pH 7.2). Membranes were washed at 52 °C in prewarmed 2× SSC/1% SDS for 10 min, followed by 1× SSC/0.5% SDS for 10 min, and 1–10 min, according to signal intensity, in 0.1× SSC/0.1% SDS. Membranes were exposed to imaging plates, and the resulting signals were visualized using a Personal Molecular Imager FX system with Quantity One software (Bio-Rad, Munich, Germany).

*Coupled Transcription/Translation Assay*: For the coupled transcription/translation assays, we employed the PURExpress^®^ In Vitro Protein Synthesis (NEB) cell-free transcription/translation system. Protein Synthesis Reaction was executed using the DHFR control template and supplemental 2.5–25 ng purified toxin Sll7039 or elution buffer respectively. Synthesis Reaction mixture was incubated at 37 °C for 2 h and reactions were stopped by placing the tubes on ice. Ten percent of the synthesis reaction were separated on 10% SDS–PAA gel electrophoresis and visualized by Coomassie-staining. The RNA of the remaining reaction mixtures was extracted by phenol/chloroform treatment followed by ethanol precipitation. RNA was separated on 10% polyacrylamide-urea gels, electroblotted onto Hybond-N+ membranes (Amersham) and cross-linked via UV-light exposure. Detection of *DHFR* mRNA transcript was performed by Northern analysis as described above.

## Figures and Tables

**Table 1:** Genomic location of TA module components re-annotated or newly defined in the course of this work. Initial and terminal positions are given according to the *Synechocystis* 6803 reference sequences for the chromosome (chr) and plasmid pSYSA (GenBank accession numbers NC_005230 and CP003267). Newly defined gene received the ID of the neighboring TA gene with the suffix ”a”. Gene *slr1062a* was previously identified as protein-coding gene *norf2* (“novel ORF 2”) based on its expression and the conserved reading frame [28] but not recognized as TA-associated. Note that all nt positions refer to the forward strand, irrespectively of the location of the respective gene. All mentioned genes were found expressed in the comparative transcriptome analysis of *Synechocystis* 6803 [29].

Gene ID	Annotation	Re-Annotation
*slr0725*	Chr:110808..111224	Chr:110727..111224
*ssr0756*	Chr:2084575..2084760	Chr:2084455..2084760
*ssr0761*	Chr:2085826..2086110	Chr:2085859..2086110
*ssl1004*	Chr:3262471..3262749	Chr:3262471..3262707
*sll1714*	Chr:968893..969294	Chr:968893..969132
*sll7006*	pSYSA:3367..3591	pSYSA:3367..3768
*ssl0350a*	Chr:2738918..2739133	novel ORF
*sll0624a*	Chr:3341880..3342128	novel ORF
*ssr0757a*	Chr:2085211..2085417	novel ORF
*ssr0761a*	Chr:2086107..2086211	novel ORF
*slr1062a*	Chr:365077..365283	novel ORF
*ssl1493a*	Chr:529033..529416	novel ORF
*ssr2784a*	Chr:3474807..3474998	novel ORF

**Table 2:** Type II TA systems of *Synechocystis* 6803 according to the TADB [16], Makarova et al. 2009 [3], more specific studies, or newly defined according to this work (TW). Systems investigated in the course of this work biochemically are labeled “BC”. The respective gene loci, protein domains, the distance between the start codon and the start site of transcription (TSS) are given. Moreover, the replicon (chr, chromosome or plasmid name pSYS A, G, M or X) and the respective arrangement of toxin (T) and antitoxin (AT) component (Ord.) are indicated. Symbols and domain names: N/K, not known; COG, Cluster of Orthologous Genes; DUF, Domain of Unknown Function; TSS, Transcriptional Start Site (R, reverse strand; F, forward strand); TU, Transcriptional Unit; asRNA, antisense RNA; PIN, PIN domain (homologues of the ”pilT N“-terminal domain); Hic, Hic domain (*hif*-contiguous); YcfA, YcfA like domain; GNAT, GCN5-related .-acetyltransferases; RelE, RelE domain (relaxed response); YhaV, YhaV domain; YoeB, YoeB domain; PemK, PemK domain (plasmid emergency maintenance); MNT, MNT domain (*minimal* nucleotidyl transferase); HigB, HigB domain (Host Inhibition of Growth); cas, CRISPR-associated; HEPN; HEPN domain (higher eukaryotes and prokaryotes nucleotide-binding); PhdYeFM-antitox, prevent-host-death protein Phd/YefM antitoxin; HTH, helix-turn-helix; VagC, VagC domain (virulence associated gene); MazE, MazE domain; AbrB, AidB regulator domain; ChpI, chromosomal homolog of pem; Xre, xenobiotic response element. Information on TSSs was taken from published studies targeting the primary transcriptome of *Synechocystis* 6803 [28,29].

Toxin	Type	A.toxin	Type	TSS	Dis.	Repl.	Ord.	Reference	Comment
*sll0205*	PIN	*ssl0385*	COG5559/DUF2281	2516776 R	27	Chr	AT-T	TW	-
*ssl0258*	HicA (YcfA)	*ssl0259*	HicB (COG1598)	2193884 R	72	Chr	T-AT	TW	only second component exhibits TSS
*sll0286*	GNAT	*sll0284*	HEPN (DUF86)	3024345 R	74	Chr	AT-T	TW	-
*ssr0335*	HicA (YcfA)	*ssr0336*	HicB (COG1598)	2754240 F	401	Chr	T-AT	TW	-
*ssl0350a*	HicA (YcfA)	*ssl0350*	HicB (COG1598)	2740742 R	1609	Chr	AT-T	TW	TSS of TU
*sll0406*	PIN	*sll0405*	hypothetical	2552996 R	34	Chr	AT-T	TW	potential asRNA regulation
*sll0525*	PIN	*ssl1004*	PhdYeFM_antitox	3262737	30	Chr	AT-T	[3,16]	see Figure 3
*sll0624*	PIN3	*sll0624a*	HicB (COG1598)	3342723 R	281	Chr	T-AT	TW	both components exhibit TSS
*sll0658*	PIN	*ssl1255*	HTH (COG2886)	3121956 R	3	Chr	AT-T	[3,16]	-
*slr0664*	RelE (DUF1044)	*ssr1114*	hypothetical	3347861 F	80	Chr	AT-T	[16,18]	-
*sll0690*	PIN	*ssl1300*	VagC	2632007 R	139	Chr	AT-T	[3,16]	alternative TSS (2631867 R)
*slr0725*	YhaV	*slr0724*	MazE (AbrB)	110366 F	26	Chr	AT-T	[3,16]	-
*ssl0739*	HicA (YcfA)	*ssl0738*	HicB (COG1598)	N/K	N/K	Chr	AT-T	TW	-
*ssr0756*	PIN	*ssr0755*	hypothetical	2084115 F	28	Chr	AT-T	TW	-
*ssr0757*	DUF497/COG2929	*ssr0757a*	DUF4415/COG3514	2084877 F	45	Chr	T-AT	TW	-
*ssr0761a*	YoeB	*ssr0761*	PhdYeFM_antitox	2085833 F	27	Chr	AT-T	TW	-
*slr0771*	PIN	*slr0770*	COG2442/Duf433	2393421 F	811	Chr	AT-T	[3,16]	joint tricistronic TU2489 with slr0769
*slr1062*	PemK	*slr1062a*	hypothetical	357724	7353	Chr	AT-T	TW	TSS of TU
*sll1092*	PIN	*ssl2138*	COG5559/DUF2281	N/K	N/K	Chr	AT-T	[16,19]	see Figure 3
*sll1130*	PemK	*ssl2245*	hypothetical	1049139 R	123	Chr	AT-T	[16,21]	potential asRNA regulation; involved in the heat shock response
*slr1210*	PIN	*slr1209*	COG2442/Duf433	891086 F	2175	Chr	AT-T	[3,16]	TSS of TU; potential asRNA regulation
*sll1225*	PIN	*ssl2420*	hypothetical	1678733 R	4435	Chr	AT-T	[16]	-
*slr1241*	MNT	*slr1240*	hypothetical	1053924 F	1650	Chr	AT-T	TW	TSS of TU
*ssr1260*	HicA (YcfA)	*ssr1258*	HicB (COG1598)	3428302 F	660	Chr	AT-T	TW	TSS of TU
*slr1327*	PIN3	*ssr2201*	hypothetical	1660041 F	28	Chr	AT-T	[16]	-
*ssl1377*	HicA (YcfA)	*ssl1376*	HicB (COG1598)	3427318 R	115	Chr	AT-T	TW	-
*slr1383*	PIN	*ssr2317*	hypothetical	679231 F	459	Chr	T-AT	TW	-
*sll1400*	PIN	*ssl2733*	COG2442/Duf433	50262 R	1627	Chr	AT-T	[3,16]	TSS of TU
*slr1421*	DUF4258	*ssr2377*	HicB (COG1598)	3273485 F	24	Chr	AT-T	TW	-
*ssl1493a*	PIN	*ssl1493*	PhdYeFM_antitox	3044721 R	66	Chr	AT-T	TW	-
*sll1504*	MNT	*sll1505*	HEPN (DUF86)	477658 R	1915	Chr	T-AT	[3,16]	TSS of TU
*sll1543*	PIN	*ssl2999*	hypothetical	3475237 R	53	Chr	T-AT	TW	both components exhibit TSS
*sll1651*	DUF497/COG2929	*sll1652*	DUF3680/COG5304	282821 R	0	Chr	T-AT	TW	leader-less
*sll1715*	PIN	*sll1714*	hypothetical	969538 R	244	Chr	AT-T	[16]	-
*ssr1766*	HicA (YcfA)	*ssr1765*	HicB (COG1598)	382338 F	54	Chr	AT-T	[3,16]	-
*slr1767*	PIN	*ssr2962*	COG2442/Duf433	544995 F	34	Chr	AT-T	[3,16,20]	alternative TSS (2085834 F); excludon-like arrangement
*slr1906*	GNAT	*slr1907*	hypothetical	609442 F	27	Chr	T-AT	TW	-
*sll1912*	PIN	*ssl3615*	hypothetical	776369 R	27	Chr	AT-T	[16]	-
*sll1965*	DUF497/COG2929	*ssl3719*	DUF4415/COG3514	915052 R	0	Chr	T-AT	TW	leader-less
*slr1999*	HicA (YcfA)	*slr1999*	HicB (COG1598)	1444698	727	Chr	AT/T	TW	both domains in one protein
*ssr2066*	HicA (YcfA)	*ssr2067*	HicB (COG1598)	1053924 F	2563	Chr	T-AT	TW	TSS of TU
*ssl2749*	MNT	*sll1411*	HEPN (DUF86)	1619636 R	1540	Chr	T-AT	[3,16]	TSS of TU
ssr2755	YoeB	*ssr2754*	PhdYeFM_antitox	2074673 F	21	Chr	AT-T	TW	-
N/K	N/K	*ssr2784*	hypothetical (ChpI homolog)	3474436 F	26	Chr	AT	TW	stand-alone
N/K	N/K	*ssr2784a*	COG2442/Duf433	N/K	N/K	Chr	AT	TW	stand-alone; potential asRNA regulation
*ssl2921*	HigB	*ssl2920*	hypothetical	N/K	N/K	Chr	AT-T	TW	TSS of TU
*ssl2923*	PIN	*ssl2922*	VagC	N/K	N/K	Chr	AT-T	[3,16]	see Figure 3
N/K	N/K	*ssr3154*	HTH (COG2886)	1211302 F	99	Chr	AT	TW	stand-alone; potential asRNA regulation
*ssr3572*	YoeB	*ssr3571*	PhdYeFM_antitox	1620247 F	29	Chr	AT-T	TW	-
*ssr3589*	PIN	*ssr3588*	hypothetical	1644850 F	3314	Chr	AT-T	TW	TSS of TU
*sll5003*	PIN	*sll5004*	COG2442/Duf433	1846 R	20	pSYSM	AT-T	[3,16]	-
*slr5017*	MNT	*slr5018*	HEPN (DUF86)	23222 F	33	pSYSM	T-AT	[3,16]	-
*ssr5020*	HigB	*slr5021*	HTH-Xre	24162 F	19	pSYSM	T-AT	[3,16]	-
N/K	N/K	*ssl5025*	COG2442/Duf433	28079 R	26	pSYSM	AT	TW	stand-alone
*sll5094*	PIN	*ssl5095*	PhdYeFM_antitox	93540 F	20	pSYSM	AT-T	[3,16]	-
*slr5102*	DUF1814	*slr5101*	hypothetical	96110 F	14	pSYSM	AT-T	TW	-
*slr5116*	PIN3	*ssr5117*	HicB	106534 F	571	pSYSM	T-AT	[3,16]	-
*slr6049*	PIN	*ssr6048*	hypothetical	47161 F	21	pSYSX	AT-T	TW	-
*slr6057*	COG2856/Duf955	*slr6056*	HTH-Xre	54326 F	17	pSYSX	AT-T	[3,16]	potential asRNA regulation; linked to possible bacterial multiubiquitin system; see Figure 4
*slr6100*	RelE (DUF2136)	*slr6101*	HTH-Xre	93754 F	66	pSYSX	T-AT	[3,16,22]	-
*sll7003*	PIN	*ssl7004*	plasmid stability	2074 R	42	pSYSA	AT-T	[3,16,23]	-
*ssl7007*	PIN	*sll7006*	HicB	3798 R	207	pSYSA	T-AT	[3,16,23]	-
*ssr7017*	cas2	*slr7016*	cas1	N/K	N/K	pSYSA	T-AT	[33]	potential asRNA regulation
*sll7030*	GNAT	*sll7031*	COG4453/DUF1778	29663 R (27264 R)	24	pSYSA	AT-T	[3,16]	-
N/K	N/K	*slr7032*	HEPN (DUF86)	30317 F (27918 F)	35	pSYSA	AT	TW	stand-alone
*sll7033*	PIN	*sll7034*	COG2442/Duf433	31387 R (28988 R)	18	pSYSA	AT-T	[3,16,23]	-
*ssl7039*	RelE	*ssl7038*	HTH-Xre	36479 R (34080 R)	0	pSYSA	T-AT	[3,16,23]	leader-less; see Figure 5 and Figure 6
*slr7041*	MazF	*ssr7040*	MazE	36567 F (34168 F)	28	pSYSA	AT-T	[3,16,23]	-
*ssl7046*	PIN3	N/K	N/K	40539 R (38140 R)	25	pSYSA	T	TW	stand-alone
N/K	N/K	*ssl7048*	MazE (AbrB)	N/K	N/K	pSYSA	AT	TW	stand-alone
*ssr7072*	*cas2*	*slr7071*	*cas1*	64765 F (62366 F)	57	pSYSA	AT-T	[33]	-
*ssr7093*	*cas2*	*slr7092*	*cas1*	88315 F	312	pSYSA	AT-T	[33]	-
*sll8007*	PIN	*ssl8008*	COG2442/Duf433	6626 R	25	pSYSG	AT-T	[3,16]	-
*sll8011*	PIN	*sll8012*	hypothetical	9650 R	29	pSYSG	AT-T	[3,16]	-
*slr8014*	PIN3	*ssr8013*	DUF2281	8658 F	1055	pSYSG	AT-T	[3,16]	see Figure 3 and Figure 7
*sll8027*	PIN3	*ssl8028*	MazE (AbrB)	24116 R	39	pSYSG	AT-T	[3,16]	-

## Supplementary Materials

The following is available online at www.mdpi.com/2072-6651/8/7/228/s1, Table S1: Comparison of toxin and antitoxin DNA sequences using blastn and a cut-off of 65% sequence identity, Table S2: Homology search of toxin and antitoxin protein sequences using blastp and a cut-off of 30% sequence identity, Table S3: Strains, Table S4: Primers.

## Abbreviations

CRISPR	clustered regularly interspaced short palindromic repeats
DUF	domain of unknown function
TA	toxin–antitoxin
TADB	toxin–antitoxin database
TSS	transcriptional start site
TU	transcriptional unit
