# Supplementary material for: Type II Toxin–Antitoxin Systems in the Unicellular Cyanobacterium Synechocystis sp. PCC 6803
Source: Toxins (Basel). 2016 Jul 21;8(7):228. doi: 10.3390/toxins8070228 (PMC4963859; doi:10.3390/toxins8070228)
Supplement: Supplementary file 1 [file toxins-08-00228-s001.pdf]

Supplementary Materials: Type II Toxin–Antitoxin Systems in the Unicellular Cyanobacterium *Synechocystis* sp. PCC 6803

Stefan Kopfmann, Stefanie K. Roesch and Wolfgang R. Hess

Table S1. Comparison of toxin and antitoxin DNA sequences using blastn and a cut-off of 65% sequence identity. Each homolog was narrowed down to one ortholog using reciprocal blastp analysis and synteny analysis of the genetic neighborhoods in PCC6803 and PCC6714.

PCC6803 Toxin	PCC6714 Homolog Present	Sequence Identity	PCC6803 Antitoxin	PCC6714 Homolog Present	Sequence Identity
sll0205	+	66.6	ssl0385	+	76
ssl0258	−	-	ssl0259	−	-
sll0286	+	86.13	sll0284	+	89
ssr0335	+	43.52	ssr0336	−	
ssl0350a	+	71.04	ssl0350	+	69.92
sll0406	−	-	sll0405	−	-
sll0525	−	-	ssl1004	−	-
sll0624	−	-	sll0624a	−	-
sll0658	+	74.25	ssl1255	+	83
slr0664	−	-	ssr1114	−	-
sll0690	−	-	ssl1300	−	-
slr0725	+	86	slr0724	+	85
ssl0739	+	74.7	ssl0738	+	88.11
ssr0756	−	-	ssr0755	−	-
ssr0757	−	-	ssr0757a	−	-
ssr0761a	−	-	ssr0761	−	-
slr0771	−	-	slr0770	−	-
slr1062	−	-	slr1062a	−	-
sll1092	−	-	ssl2138	−	-
sll1130	+	91	ssl2245	+	93
slr1210	−	-	slr1209	−	-

sll1225	+	83.16	ssl2420	+	68.8
slr1241	−	-	slr1240	−	-
ssr1260	−	-	ssr1258	−	-
slr1327	−	-	ssr2201	−	-
ssl1377	−	-	ssl1376	−	-
slr1383	−	-	ssr2317	−	-
sll1400	−	-	ssl2733	−	-
slr1421	+	68.73	ssr2377	+	89.28
ssl1493a	−	-	ssl1493	−	-
sll1504	+	83.6	sll1505	+	65.12
sll1543	−	-	ssl2999	−	-
sll1651	−	-	sll1652	−	-
sll1715	−	-	sll1714	−	-
ssr1766	−	-	ssr1765	−	-
slr1767	−	-	ssr2962	−	-
slr1906	+	82.17	slr1907	+	78
sll1912	+	69.42	ssl3615	+	57.51
sll1965	−	-	ssl3719	−	-
slr1999	−	-	slr1999	−	-
ssr2066	−	-	ssr2067	−	-
ssl2749	−	-	sll1411	−	-
ssr2755	−	-	ssr2754	−	-
N/K	−	-	ssr2784	−	-
N/K	−	-	ssr2784a	−	-
ssl2921	+	96	ssl2920	+	94
ssl2923	+	89	ssl2922	+	77.22
N/K	-	-	ssr3154	−	-
ssr3572	−	-	ssr3571	−	-
ssr3589	−	-	ssr3588	−	-
sll5003	−	-	sll5004	−	-
slr5017	−	-	slr5018	−	-
ssr5020	−	-	slr5021	−	-

N/K	−	-	ssl5025	−	-
sll5094	+	91	ssl5095	+	90
slr5102	−	-	slr5101	−	-
slr5116	+	89	ssr5117	+	94
slr6049	−	-	ssr6048	−	-
slr6057	−	-	slr6056	−	-
slr6100	−	-	slr6101	−	-
sll7003	+	93	ssl7004	+	95
ssl7007	+	95	sll7006	+	91.08
ssr7017	−	-	slr7016	−	-
sll7030	−	-	sll7031	−	-
N/K	-	-	slr7032	−	-
sll7033	−	-	sll7034	−	-
ssl7039	−	-	ssl7038	−	-
slr7041	−	-	ssr7040	−	-
ssl7046	−	-	N/K	−	-
N/K	-	-	ssl7048	−	-
ssr7072	−	-	slr7071	−	-
ssr7093	+	96	slr7092	+	97
sll8007	−	-	ssl8008	−	-
sll8011	−	-	sll8012	−	-
slr8014	−	-	ssr8013	−	-

Table S2. Homology search of toxin and antitoxin protein sequences using blastp and a cut-off of 30% sequence identity. Each homolog was narrowed down to one ortholog using reciprocal blastp analysis and synteny analysis of the genetic neighborhoods in PCC6803 and PCC6714.

**PCC6803**	**PCC6714**		**PCC6803**	**PCC6714**	
**Toxin**	**Homolog**	**Homolog**	**Antitoxin**	**Homolog**	**Homolog**
	**Blastp**	**Reciprocal Blastp + Synteny**		**Blastp**	**Reciprocal Blastp + Synteny**
sll0205	D082_05270	D082_05270	ssl0385	D082_05260	D082_05260
ssl0258	D082_50470	-	ssl0259	D082_50460	-
sll0286	D082_22430	D082_22430	sll0284	D082_22420	D082_22420
ssr0335	D082_30800	-	ssr0336	D082_30810	-

ssl0350a	D082_30800	D082_30800	ssl0350	D082_30810/D082_27220	D082_30810
sll0406	-	-	sll0405	-	-
sll0525	D082_40900	D082_40900	ssl1004	D082_40890	D082_40890
sll0624	D082_60470	D082_60470	sll0624a	D082_60460	D082_60460
sll0658	D082_03850/D082_03860; D082_60140	D082_03850/D082_03860; D082_60140	ssl1255	D082_03840; D082_60140	D082_03840; D082_60140
slr0664	-	-	ssr1114	-	-
sll0690	D082_30840	-	ssl1300	D082_30830	-
slr0725	D082_50930	D082_50930	slr0724	D082_50940	D082_50940
ssl0739	D082_07310	D082_07310	ssl0738	D082_07300	D082_07300
ssr0756	D082_40900/D082_05270	-	ssr0755	D082_05260	-
ssr0757	-	-	ssr0757a	-	-
ssr0761a	-	-	ssr0761	-	-
slr0771	-	-	slr0770	D082_15400	D082_15400
slr1062	D082_17650	D082_17650	slr1062a	D082_17660	D082_17660
sll1092	D082_05270	-	ssl2138	D082_05260	-
sll1130	D082_34540	D082_34540	ssl2245	D082_34530	D082_34530
slr1210	-	-	slr1209	D082_15400	-
sll1225	D082_26250	D082_26250	ssl2420	D082_26250a	D082_26250a
slr1241	-	-	slr1240	D082_34440	D082_34440
ssr1260	D082_50470	-	ssr1258	D082_50460	-
slr1327	D082_40860	D082_40860	ssr2201	-	-
ssl1377	-	-	ssl1376	-	-
slr1383	D082_28260	D082_28260	ssr2317	D082_28250	D082_28250
sll1400	D082_41030	D082_41030	ssl2733	D082_41020	D082_41020
slr1421	-	-	ssr2377	D082_13570	D082_13570
ssl1493a	-	-	ssl1493	-	-
sll1504	D082_30880/D082_3900	D082_30880/D082_3900	sll1505	D082_30890	D082_30890
sll1543	-	-	ssl2999	-	-
sll1651	-	-	sll1652	-	-
sll1715	-	-	sll1714	-	-
ssr1766	D082_50470	D082_50470	ssr1765	D082_50460	D082_50460
slr1767	D082_41030	-	ssr2962	D082_41020	-
slr1906	D082_04370	D082_04370	slr1907	D082_04380	D082_04380
sll1912	D082_12830	D082_12830	ssl3615	-	-
sll1965	-	-	ssl3719	-	-
slr1999	-	-	-	-	-

ssr2066	-	-	ssr2067	D082_34430	D082_34430
ssl2749	D082_40370	D082_40370	sll1411	D082_40380	D082_40380
ssr2755	D082_30870	-	ssr2754	-	-
N/K	-	-	ssr2784	-	-
N/K	-	-	ssr2784a	-	-
ssl2921	D082_30870	D082_30870	ssl2920	D082_30860	D082_30860
ssl2923	D082_30840	D082_30840	ssl2922	D082_30830	D082_30830
N/K	-	-	ssr3154	-	-
ssr3572	D082_30870	-	ssr3571	-	-
ssr3589	-	-	ssr3588	-	-
sll5003	-	-	sll5004	-	-
slr5017	D082_30880/D082_3900;	-	slr5018	D082_30890	-
ssr5020	D082_30440a	D082_30440a	slr5021	D082_30440	D082_30440
N/K	-	-	ssl5025	D082_08740	D082_08740
sll5094	D082_50890	D082_50890	ssl5095	D082_50880	D082_50880
slr5102	-	-	slr5101	-	-
slr5116	D082_50980	D082_50980	ssr5117	D082_50970	D082_50970
slr6049	D082_50890	-	ssr6048	-	-
slr6057	-	-	slr6056	-	-
slr6100	-	-	slr6101	-	-
sll7003	D082_40020	D082_40020	ssl7004	D082_40010	D082_40010
ssl7007	D082_60470	D082_60470	sll7006	D082_60460	D082_60460
ssr7017	D082_40660	D082_40660	slr7016	D082_40020	-
sll7030	-	-	sll7031	-	-
N/K	-	-	slr7032	D082_30890	-
sll7033	-	-	sll7034	D082_15400	-
ssl7039	-	-	ssl7038	-	-
slr7041	-	-	ssr7040	-	-
ssl7046	-	-	N/K	-	-
N/K	-	-	ssl7048	-	-
ssr7072	-	-	slr7071	-	-
ssr7093	D082_40660	D082_40660	slr7092	D082_40670	D082_40670
sll8007	-	-	ssl8008	-	-
sll8011	-	-	sll8012	-	-
slr8014	-	-	ssr8013	-	-
sll8027	-	-	ssl8028	-	-

Table S3. Strains.

E. coli Strains	Description	Source
**TOP10 F′**	F′{*lac*Iq, Tn*10*(TetR)} *mcr*A Δ(*mrr-hsd*RMS-*mcr*BC) Φ80*lac*ZΔM15 Δ*lac*X74 *rec*A1 *ara*D139 Δ(*ara leu*) 7697 *gal*U *gal*K *rps*L (StrR) *end*A1 *nup*G	Invitrogen
**M15 [pREP4]**	nal^S str^S rif^S thi^- lac^- ara^+ gal^+ mtl^- f^- $recA^+$ uvr^+ lon^+	Quiagen
**BL21 (DE3) Rosetta™**	F^- *omp*T *gal dcm lon hsd*SB(r_{B}^- m_{B}^-) λ(DE3 [*lac*I *lac*UV5-T7 gene 1 *ind*1 *sam*7 *nin*5]) [*argU ileY leuW* Cam^r]	Novagen

Table S4. Primers.

Primer	Sequence
**sll0525_SphI_FW**	5′-AGAAGGATTAGCATGCAACTTTTGTTGG
**sll0525_BamHI_RV**	5′-TGGCGATCGACTCTGGATCCTACTCC
**sll1092_SphI_FW**	5′-AGGAGTATCTGGCATGCATTCTGTTTTG
**sll1092_BamHI_RV**	5′-AGGTCGCTATGGATCCCAACCACAG
**ssl2923_SphI_FW**	5′-GAGAAGAGTTCTGCATGCAGTATTTACTAG
**ssl2923_BamHI_RV**	5′-TACTTGGATCCTTGCCATAGGCGATCG
**ssl1004_SacI_FW**	5′-CGATGAGAGAGCTCTATGATCGGAG
**ssl1004_KpnI_RV**	5′-GTTTCACGTTAAGGTACCTCAAGATAATGGCCA
**ssl2138_SacI_FW**	5′-CATTAAATTCAAGGAGAGCTCAATGACTACTG
**ssl2138_KpnI_RV**	5′-CAAAACAGGGTACCTTACATATACTCCTC
**ssl2922_SacI_FW**	5′-TTACTTATCCTAGAGGGAGCTCCATGAACAC
**ssl2922_KpnI_RV**	5′-GTATCTAGTAAATAGGTACCTTAGAACTCTTCTCG
**ssr8013_SacI_FW**	5'-GGTTGAGCTCCATGACAGCG
**ssr8013_KpnI_RV**	5'-GTACTTTCACAGGTACCTTACAGATACTTCTC
**slr8014_NdeI_FW**	5'-GTATCTGTAAACATATGGTGAAAGTACTTC
**slr8014_XhoI_RV**	5'-ATGGGCCTCGAGATCAAGCTTATTG
**ssl7038_SacI-fw**	5'-AGAACTATAAGGTGAGCTCAATGGAAACCC
**ssl7038_EcoRI-rv**	5'-AACATTACCTGAATTCCGAGTGTCGTGG
**ssl7039_SphI-fw**	5'-GGATACAATAGGGGCATGCTTGAAATCC
**ssl7039_BamHI-rv**	5'-TGAATTGGATCCATAGTTCTTTGGCTAAATC
**lpp_T7_TS_RV**	5'TAATACGACTCACTATAGGGACAGAAGACAGCTGATCGATTTTAG
**lpp_TS_FW**	5'-AATGAAAGCTACTAAACTGGTACTGG
**DHFR P1-T7_RV**	5'-TAATACGACTCACTATAGGGAAAACCAGGCGAGATCGG
**DHFR P1_FW**	5'-ATATACATATGATCAGTCTGATTGC
**DHFR Plasmid seq_FW**	5′-GCTAGTGGTGCTAGCCC
**DHFR Plasmid seq_RV**	5′-CAAGCTTCCGGATATAGTTCC
